# Overexpression of *Liriodendron* Hybrid *LhGLK1* in *Arabidopsis* Leads to Excessive Chlorophyll Synthesis and Improved Growth

**DOI:** 10.3390/ijms25136968

**Published:** 2024-06-26

**Authors:** Haoxian Qu, Shuang Liang, Lingfeng Hu, Long Yu, Pengxiang Liang, Zhaodong Hao, Ye Peng, Jing Yang, Jisen Shi, Jinhui Chen

**Affiliations:** 1 State Key Laboratory of Tree Genetics and Breeding, Co-Innovation Center for Sustainable Forestry in Southern China, Key Laboratory of Forest Genetics & Biotechnology of Ministry of Education, Nanjing Forestry University, Nanjing 210037, China; qhx1881@163.com (H.Q.); shuanglliang@163.com (S.L.); hlf625@njfu.edu.cn (L.H.); 15957571016@163.com (L.Y.); lpx20021@163.com (P.L.); haozd@njfu.edu.cn (Z.H.); 2 College of Life Sciences, Nanjing Forestry University, Nanjing 210037, China; pengye@njfu.edu.cn; 3 Advanced Analysis and Testing Center, Nanjing Forestry University, Nanjing 210037, China; yjnjfu@sina.com

**Keywords:** *LhGLK1*, ectopic chloroplasts biogenesis, chlorophyll, photosynthesis, *Liriodendron* hybrid

## Abstract

Chloroplasts is the site for photosynthesis, which is the main primary source of energy for plants. Golden2-like (GLK) is a key transcription factor that regulates chloroplast development and chlorophyll synthesis. However, most studies on *GLK* genes are performed in crops and model plants with less attention to woody plants. In this study, we identified the *LhGLK1* and *LhGLK2* genes in the woody plant *Liriodendron* hybrid, and they are specifically expressed in green tissues. We showed that overexpression of the *LhGLK1* gene improves rosette leaf chlorophyll content and induces ectopic chlorophyll biogenesis in primary root and petal vascular tissue in *Arabidopsis*. Although these exhibit a late-flowering phenotype, transgenic lines accumulate more biomass in vegetative growth with improved photochemical quenching (qP) and efficiency of photosystem II. Taken together, we verified a conserved and ancient mechanism for regulating chloroplast biogenesis in *Liriodendron* hybrid and evaluated its effect on photosynthesis and rosette biomass accumulation in the model plant *Arabidopsis*.

## 1. Introduction

Chloroplasts are semi-autonomous organelles of plants whose biogenesis depends on the co-expression of nuclear and plastid genes [1]. It is widely believed that the origination of chloroplasts followed an endosymbiotic event between a photosynthetic cyanobacterium and a eukaryote [2]. In the process of evolution, most genes in cyanobacteria were gradually transferred to the host genome [3]. With the germination of seeds, chloroplasts are originally transformed from proplastids, which are colorless organelles without chlorophyll or photosynthesis [3]. As signals, light and plant hormones participate in the biogenesis of chloroplasts [4]. Subsequently, nuclear-coded photosynthetic components are synthesized in the cytoplasm and imported to the chloroplast, which are precisely targeted to protein complexes in photosystems and thylakoids that are coded by the plastid genome. The mechanism of regulating this process has been described before [3,5]. In addition, during the lifetime of a plant, variation in sunlight intensity and stress may have an impact on chloroplast biogenesis. After a long evolution, plants have formed a regulatory network to adapt to changes in the environment [6]. By accepting retrograde signals from the plastid, plants coordinate nuclear photosynthetic gene expression and growth conditions to sustain the normal function of chloroplasts [6]. For most photosynthetic plants, only a few transcription factors that function in regulating plant chloroplast biogenesis have been reported [7,8]. Over the past few decades, research on the nuclear transcription factor GLK has shown that GLK plays an important role in regulating photosynthesis-associated nuclear genes and then promotes chloroplast biogenesis [9]. 

*GLK* (*Golden2-like*) genes were first discovered and named in the research of maize mutant in 1920s and were found only in plants [10,11,12]. *GLK* genes belong to the GARP super gene family, which is named after the maize *GOLDEN2* gene, *Arabidopsis ARR* B-class genes, and *Chlamydomonas PSR1* [13,14]. Besides their function in regulating chloroplast biogenesis, as mentioned before, other studies indicated that *GLK* genes also participate in biotic or abiotic stress and leaf senescence [6,15,16,17]. Based on the results of evolutionary analysis, *GLK* genes existed before the evolution of land plants [2]. A typical GLK protein structure has two conserved domains: the N-terminal DNA binding domain (DBD) and the C-terminal GCT-box [1,18,19]. The DNA binding domain is responsible for binding to downstream target genes, and the DBD domain is sufficient to promote reporter gene transcription in the yeast transactivation trail [13]. GCT-box may play a role in homo- and heterodimerization of the maize G2 and ZmGLK1 proteins, both of which both may be necessary to conduct its function in vivo [13]. GLK proteins are highly conserved in two domains in different species. *GLK* genes promote chloroplast formation by binding to downstream promoters of photosynthesis-related genes and driving their transcription, which mainly contain genes coding key enzymes in chlorophyll biosynthesis and protein subunits in the photosystem such as HEMA1, GUN4, PORA, CAO, Lhca, Lhcb [20,21]. A previous study of Arabidopsis showed that the target genes of AtGLKs are mainly related to chlorophyll synthesis and light harvesting [20]. Notably, photosynthesis-related genes are the most highly represented and significantly enriched in overexpressing *AtGLK* lines [20]. Even though the function of GLK1 and GLK2 is highly redundant in Arabidopsis, the differential expression gene sets are slightly different between *atglk1* and *atglk2* in transcriptome data [20]. In the C4 plant maize, a tissue-specific expression pattern has been observed, which is relative to photosynthetic cell differentiation [13]. Moreover, mutation of *ZmG2* impeded the chloroplast biogenesis in bundle sheath cells. In the latest study of maize, *ZmGLK1* was not required for chloroplast biogenesis in mesophyll cells. These data showed that although genome replication events lead to the replication of *GLK* genes, the function of *GLK* genes may have been partly diverged.

In *Arabidopsis*, the loss of two *GLK* genes leads to incompletely developed chloroplasts, lower chlorophyll content, and light green phenotype in leaves [1,3]. On the contrary, overexpression of *GLK* genes will promote chlorophyll biosynthesis and thylakoid stacking in many species, such as Arabidopsis, tomato, rice [22,23,24,25]. Interestingly, *GLK* genes have an ability to enhance chloroplast biogenesis in non-green tissues. Chloroplasts were observed and tested in overexpressing *GLK* rice callus and *Arabidopsis* roots [4,26]. In tomatoes, fruit showed a dark-green phenotype with more chlorophyll content and nutrition accumulation by overexpressing *SlGLK* genes [23,27]. In addition, GLK protein function is highly conserved in different species. The expression of moss *PpGLKs* can partly save the phenotype of the double mutant *glk1glk2* in Arabidopsis, which is the most distantly species to the angiosperms [2,28]. Even relative research showed that some components of the GLK regulation network evolved independently in different species; this ancient and conserved mechanism may have existed for at least 400 million years and began to evolve before land plants [28,29].

In recent years, with the increasing requirements for agriculture and forestry, studies about *GLK* genes have shown their great potential in agriculture and forestry breeding. More chlorophyll content and chloroplasts may change the photosynthetic characteristics of plants. Overexpression of *ZmGLKs* enhanced the photosynthetic rate of rice with increased grain yield. Nevertheless, some studies revealed that excessive chloroplasts and chlorophyll are unfavorable to normal photosynthesis [24]. In some cases, the effect of overexpressing *ZmGLKs* in rice will appear when plants are cultured under strong light, while no marked difference under moderate light conditions were found [22]. In *Arabidopsis*, negative results regarding photosynthesis and biomass accumulation were observed in overexpressing *AtGLKs* lines as transgenic lines showed less rosette biomass accumulation with decreased qP and ΦPSII [7]. These studies indicated that using *GLK* genes to improve the photosynthetic characteristics of plants is complicated but worthy.

*Liriodendron* hybrid was obtained by artificial hybridization, and its parents are *Liriodendron chinense* and *Liriodendron tulipifera*. The genus *Liriodendron* is an ancient relic group with an evolutionary position between angiosperms and eudicots. The *Liriodendron* hybrid is famous for timber and ornamental value in China due to its strong heterosis [30,31]. There are many studies about *GLK* genes in many crops and *Arabidopsis*. To our knowledge, there are few relevant studies on woody plants besides Birch [32]. By establishing somatic embryogenesis and transgenesis systems, *Liriodendron* hybrid shows great potential in production and breeding [32]. Here, we identified *LhGLK1* and *LhGLK2*, and overexpressed *LhGLK1* in Arabidopsis. Our data confirmed that LhGLKs have highly conserved DBD and GCT domains, in keeping with Arabidopsis and crops. Not surprisingly, the function of LhGLK1 in regulating chlorophyll biosynthesis and chloroplast formation was also observed in Arabidopsis. In addition, vegetative growth and parameters of photosynthesis were improved when *LhGLK1* was overexpressed in Arabidopsis. We expand the boundaries of studies about GLK based on crops and explore the possibility of *LhGLK1* in improving plant photosynthesis. We present a vision that the application of GLK in woody plants and crops may bring more biomass accumulation and CO_2_ absorption with increased photosynthetic intensity and decreased photoinhibition [33,34]. 

## 2. Results

### 2.1. Identification and Tissue Expression Pattern Analysis of LhGLKs

**Figure 1:**
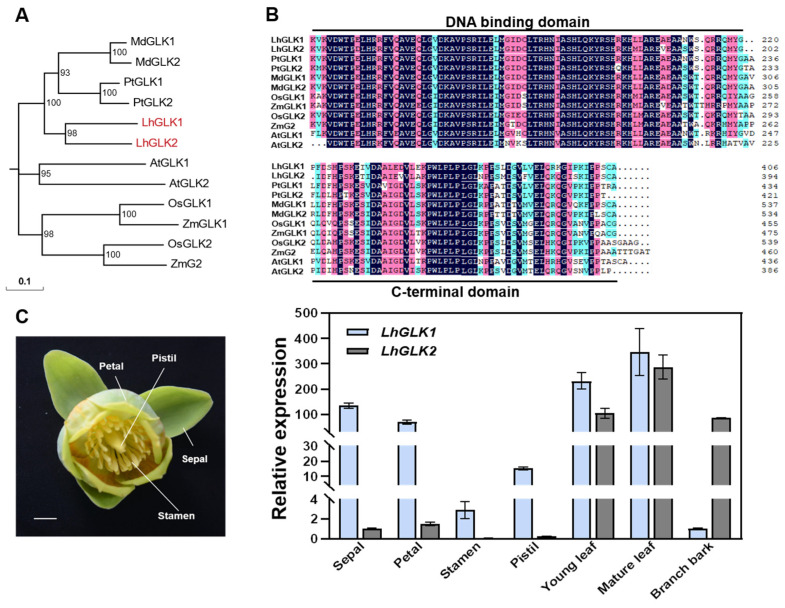
Identification and expression analysis of *LhGLKs.* (**A**) Phylogenetic analysis of GLK1 and GLK2 from *Liriodendron hybrid* (Lh), *Arabidopsis thaliana* (At), *Oryza sativa* (Os), *Zea mays* (Zm), *Malus domestica* (Md) and *Populus tomentosa* (Pt). The tree was constructed on the software MEGA7, using neighbor-joining (NJ) method with 1000 bootstrap replicates. (**B**) Amino acid sequence alignment of GLK genes from six species. The black lines represent the conserved DNA binding domain and the C-terminal domain. (**C**) Relative expression analysis of *LhGLK1* and *LhGLK2* in different tissues of *Liriodendron* hybrid. Three biological replicates of each tissue were harvested for qRT-PCR experiments. Error bars mean the SD of three biological replicates. Scale bar = 1 cm.

To identify GLK1 and GLK2 in the *Liriodendron* hybrid, we used the GLK1 and GLK2 protein sequences from *Arabidopsis thaliana*, *Oryza sativa*, *Zea mays*, *Malus domestica* and *Populus tomentosa* as queries to blast against the protein database of *Liriodendron chinense*, and then designed primers to clone *LhGLK* genes in *Liriodendron* hybrid seedlings. The amino acid sequences translated from CDS were aligned with GLK genes of the mentioned species, finding that they contained the conserved DNA binding domain and the C-terminal domain (Figure 1B). Next, a phylogenetic analysis suggested that LhGLKs are more closely related to GLK genes in poplar and apple (Figure 1A). 

Previous studies demonstrated that the *GLK1* and *GLK2* genes are specifically expressed in green tissues and have different expression patterns. To confirm that, we collected floral organs, leaves, and branch bark from the mature tree and leaves from seedlings of *Liriodendron* hybrid. The results of real-time quantitative PCR (qRT-PCR) showed that the expression levels of *LhGLKs* are higher in green tissues such as sepals, petals and leaves. Notably, *LhGLK2* is only specifically expressed in leaves and branch bark (Figure 1C). 

### 2.2. Overexpression of LhGLK1 in Arabidopsis Leads to More Chlorophyll Accumulation and Promotes Chloroplast Formation in Non-Green Tissue

**Figure 2:**
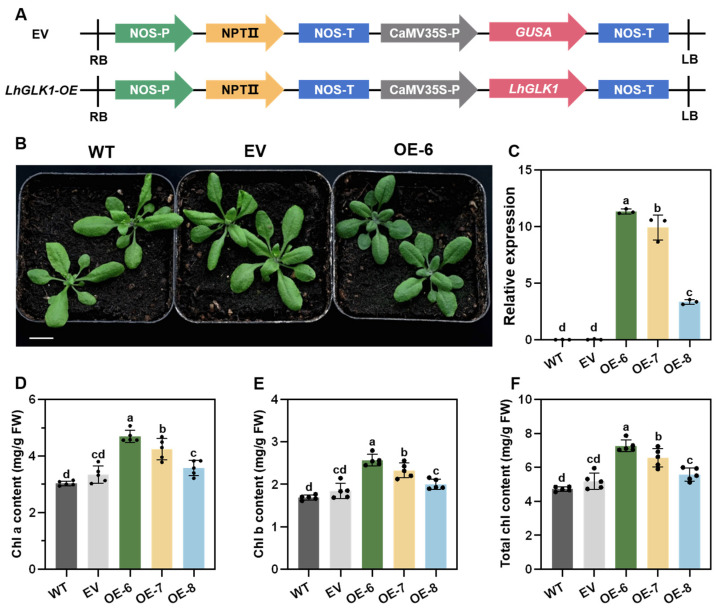
Overexpression of *LhGLK1* in Arabidopsis promotes Chlorophyll biosynthesis. (**A**) Schematic diagram of the *LhGLK1* overexpression (*LhGLK1-OE*) vector with CaMV35S promotor and empty vector (EV). (**B**) The phenotype of dark green leaves in the line #6 of overexpressing *LhGLK1*, comparing to wild-type (WT) and empty vector (EV). Scale bar = 1 cm. (**C**) Relative expression levels of three different *LhGLK1-OE* lines. The vertical axis values represent 2^(−ΔCT)^, and the average *±* SE is shown (*n* = 3 biological replicates). Different letters over the bar represent significant differences based on one-way ANOVA tests (*p* < 0.05). (**D**–**F**) Chlorophyll a content (**D**), chlorophyll b content (**E**), and total chlorophyll content (**F**) (mg/g of fresh weight) in leaves of WT, EV, and three *LhGLK1-OE* lines (OE6, OE7 and OE8). All the values are means *±* SE (*n* = 5 biological replicates). The letters a, b, c and d over the bar represent significant differences based on one-way ANOVA tests (*p* < 0.05), with groups marked by identical letters having no significant difference between them.

GLK is a key component in regulating the formation of chloroplasts. Defective chloroplasts were observed in the *glk1 glk2* double mutant of Arabidopsis [20]. Here, we generated overexpressed *LhGLK1* lines in Arabidopsis, which were driven by the 35S cauliflower mosaic virus promoter (Figure 2A). Then, we examined the transcription level of *LhGLK1* in leaves by qRT-PCR. All three transgenic lines showed different overexpression levels compared to control lines (Figure 2C). Rosette leaves of transgenic plants showed a dark-green phenotype after being cultured on soil (Figure 2B and Figure S1). We visualized and decomposed the vision disparity into L, a, b aspects by CIE (Figure S2). Value L, a and b represent color brightness, red-green and yellow-blue, respectively. All three transgenic lines have a decreased absolute value at L, a and b when compared with control lines, which means transgenic lines show dark-green. We believe that the reason for the dark-green phenotype of leaves is due to excessive accumulation of chlorophyll in transgenic lines; so, the total chlorophyll of rosette leaves was extracted and measured. Consistent with dark-green phenotype, chlorophyll content was much higher in OE lines (Figure 2D–F). Because chlorophyll a and b absorb light at different wavelengths, the chlorophyll a/b ratio is a signature of photosynthetic apparatus state; so, the Chl a/b ratio was calculated, which showed no remarkable change in transgenic lines (Figure S3).

**Figure 3:**
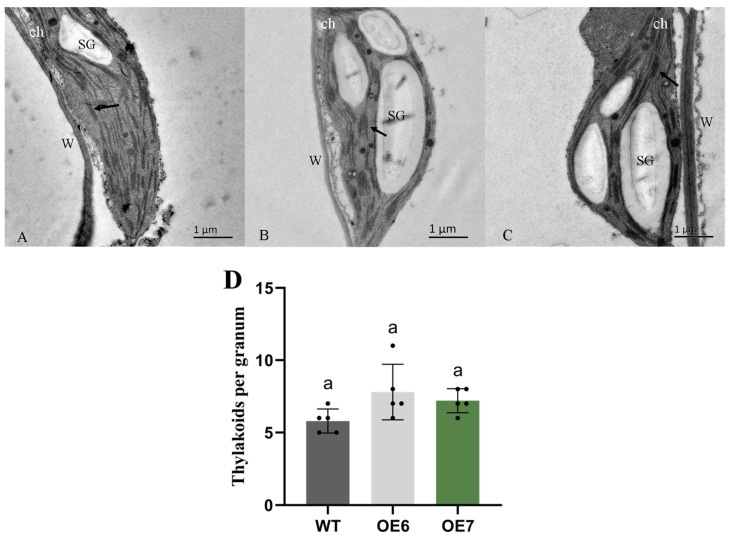
Transmission electron microscopy (TEM) of leaf chloroplast ultrastructure in (**A**) wild-type and (**B**) OE-6 and (**C**) OE-7. ch: chloroplast; W: wall; SG: starch granule. The arrow points to the thylakoid. (**D**) Quantification of granal stacking in mesophyll cells. Thylakoids were counted in chloroplast of 5 different cells. Scar bar = 1 μm. All the values are means *±* SE (*n* = 5 biological replicates). The letters over the bar represent significant differences based on one-way ANOVA tests (*p* < 0.05), with groups marked by identical letters having no significant difference between them.

In order to study the effect of *LhGLK1* overexpression on chloroplast biosynthesis, we observed the ultrastructure of leaf chloroplast by transmission electron microscopy. As shown in Figure 3, the chloroplast of OE lines exhibits a higher thylakoid number, but no significant difference was found in comparison to WT.

**Figure 4:**
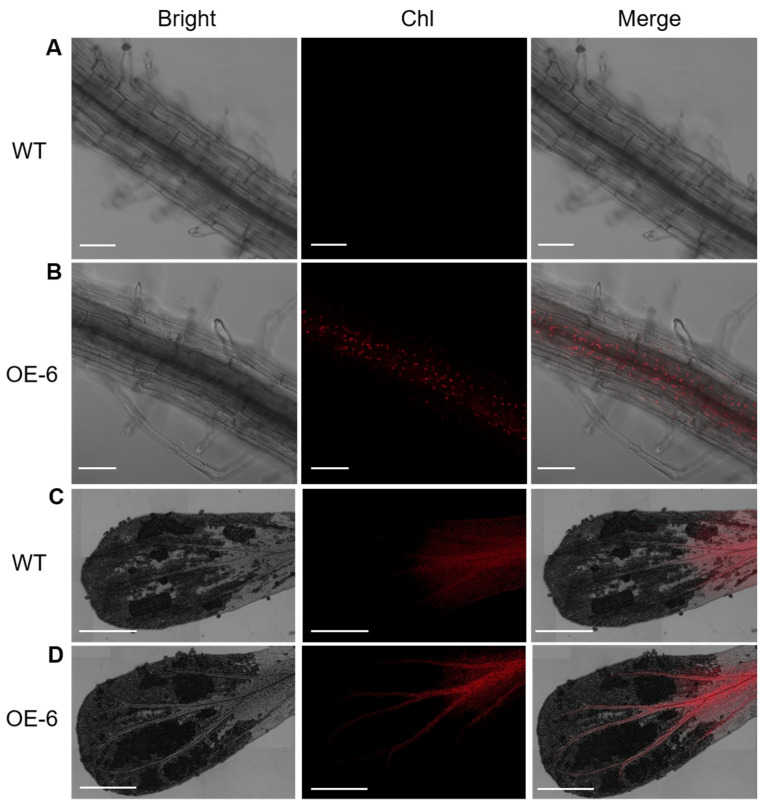
Overexpression of *LhGLK1* leads to ectopic chlorophyll biosynthesis in primary root and petal vascular tissue in Arabidopsis. (**A**,**B**) The chlorophyll (Chl) autofluorescence confocal micrographs of the primary root in wild-type and LhGLK1-OE6 seedlings, respectively. Scale bar = 50 µm. (**C**,**D**) The chlorophyll autofluorescence confocal micrographs of the petal in wild-type and LhGLK1-OE6 Arabidopsis lines, respectively. Scale bar = 1 mm. All the Chl micrographs above was scanned under 488 nm laser excitation.

Previous studies about GLK genes in Arabidopsis showed that GLK genes have the capacity to induce chloroplast formation in roots. Not all cells have the potential to form a chloroplast, and it is considered that non-photosynthetic cells are regulated by other factors to prevent the formation of chloroplast [3]. In this study, chlorophyll autofluorescence of experimental plants was conducted and observed by confocal microscope [35]. Consistent with previous information, there is strong chlorophyll autofluorescence in the middle of primary roots of OE lines (Figure 4A,B). At the same time, WT and EV lines showed no fluorescence signals in root cells, regardless of cell type. Similar cases were observed in petals of transgenic lines. Although chlorophyll autofluorescence was present in the bottom of the petal in all lines, the fluorescence signal disappeared suddenly in the upper vascular bundle of the petal in WT and EV lines. On the contrary, the fluorescence signal was still sustained in the upper vascular bundle of the petal in transgenic lines (Figure 4C,D). 

**Figure 5:**
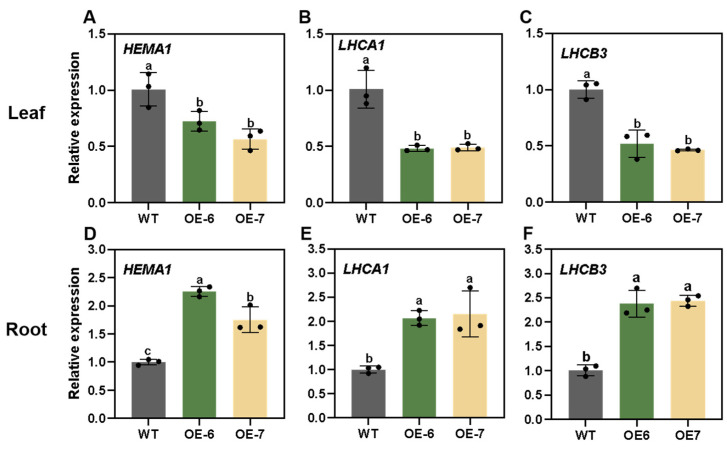
Relative expression levels of chloroplast biogenesis and chlorophyll synthesis genes in leaves (**A**–**C**) and roots (**D**–**F**) of wild-type and *LhGLK1* overexpression lines analyzed by qRT-PCR. All the values are means ± SE (*n* = 3 biological replicates). Different letters over the bar represent significant differences based on one-way ANOVA tests (*p* < 0.05), with groups marked by identical letters having no significant difference between them..

**Figure 6:**
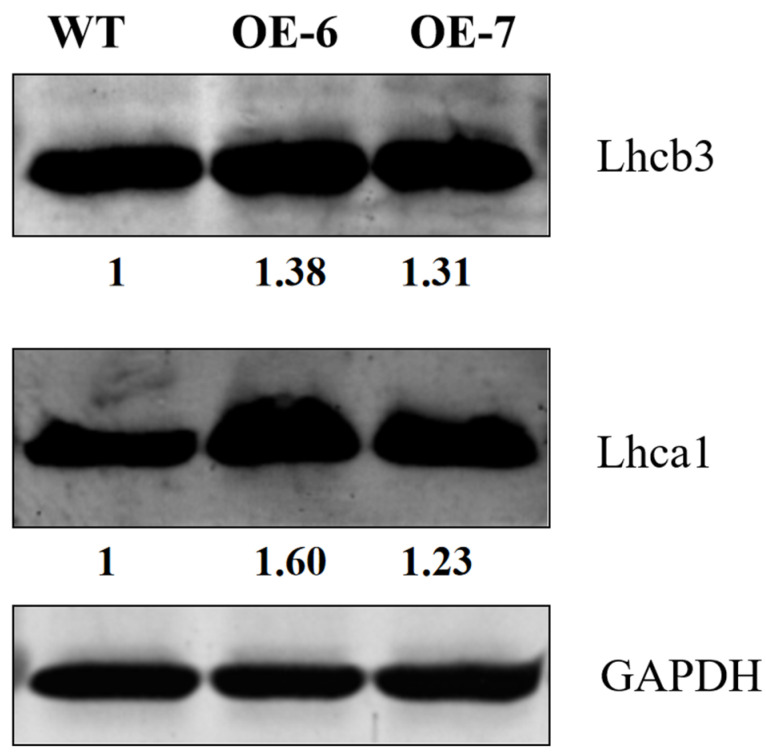
Western blot analysis of photosynthetic proteins. Photosynthetic proteins in 10 mg of total membrane protein from leaf samples of wild type and OE lines. The values represent relative protein content to GAPDH.

Furthermore, we tested the transcription levels of *LhGLK1* and GLK target genes in roots by qRT-PCR. As expected, the expression levels of GLK target genes were up-regulated compared to those of WT (Figure 5D–F). Nevertheless, the expression levels of the mentioned genes were down-regulated in leaves (Figure 5A–C). Conversely, Western blot shows that there are more Lhca1 and Lhcb3 proteins in OE leaf (Figure 6). Increased protein levels may reduce the expression of *LHCA1* and *LHCB3*. Enhanced expression of photosynthesis-associated nuclear genes may contribute to the biogenesis of chlorophyll in the root. In conclusion, the above data are consistent with GLK genes’ function, which positively regulate chloroplast and chlorophyll biosynthesis in green and some specific non-green tissues.

### 2.3. LhGLK1 Overexpression Changes Photosynthetic Characteristics and Flowering Time and Improved Growth 

**Figure 7:**
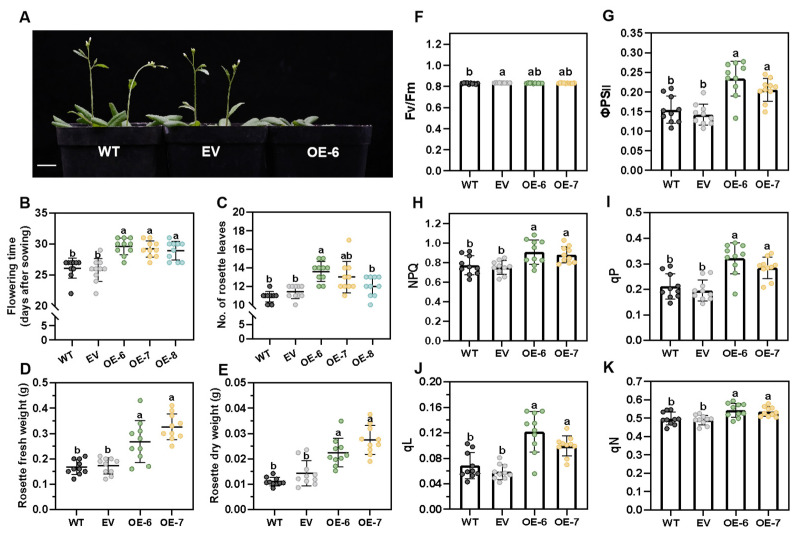
Overexpressing LhGLK1 changed photosynthetic characteristics and delayed flowering time of Arabidopsis and affected its growth. (**A**) The late-flowering phenotype of *LhGLK1-OE* lines compared to wild-type and empty vector control Arabidopsis. Scale bar = 1 cm. (**B**) The flowering time (as days after sowing) of WT, EV and three OE lines. (**C**) The flowering time (as number of rosette leaves) of WT, EV and tree OE lines. (**D**) The rosette fresh weight of WT, EV and two OE lines. (**E**) The rosette dry weight of WT, EV and two OE line. (**F**–**K**) The chlorophyll fluorescence characteristics of WT, EV and OE lines. Different letters over the bar represent significant differences based on one-way ANOVA tests (*p* < 0.05), with groups marked by identical letters having no significant difference between them.

Improving photosynthetic efficiency was regarded as a viable way to enhance grain yield potential in crops [22]. Many studies focusing on the influence of *GLK* on plant photosynthetic characteristics and grain field have been conducted. Excessive chlorophyll and chloroplast may have impacts on plant photosynthetic intensity and photoinhibition. In our study, we measured chlorophyll fluorescence parameters of experimental plants to analyze the variations in photosynthetic characteristics in transgenic lines. As shown in the figure, the efficiency of photosystem II (ΦPSII) is enhanced in OE lines, which means OE lines are more efficient in using energy for photosynthesis (Figure 7G). At the same time, photochemical quenching (qP) and qL were significantly increased in OE lines, which shows that more energy was used for photosynthesis instead of heat (Figure 7I,J). Nevertheless, we did not observe any significant changes in maximum quantum efficiency of photosystem II (Fv/Fm) (Figure 7F). In addition, parameter non-photochemical quenching (NPQ) and non-photochemical quenching (qN) represent the fraction of regulated heat dissipation. NPQ and qN are slightly higher in OE lines (Figure 7H,K). This parameter implies the ability of photoprotection.

Overexpressing *AtGLK1* in Arabidopsis exhibited late-flowering phenotype, whose number of rosette leaves and flowering time delay were significantly increased compared to WT [7]. As with the previous note, flowering time delay was observed in OE lines with more rosette leaf number and rosette biomass accumulation (Figure 7A–C). In addition, OE lines exhibit higher fresh and dry weight of rosette leaves compared to the control lines (Figure 7D,E).

## 3. Discussion 

Chloroplast biogenesis in land plants is critical and necessary for the ecological system. Nearly all energy in the ecosystem comes from photosynthesis. Normal chloroplast biogenesis relies on photosynthesis-associated nuclear and plastid genes. Some researchers deem that there are main controllers to regulate chloroplast biogenesis in plants [7,36]. Recently, transcription factor GLK and GNC showed great function in regulating chloroplast biogenesis. Due to woody plants having longer life cycles and more difficult transgenesis systems, few experiments were performed. Here, we found that GLK proteins have conserved DBD and GCT domains, regardless of whether in crops or woody plants. We then mainly generated ectopic overexpression of *LhGLK1* lines to verify its function in promoting chloroplast formation and evaluating its improvement to photosynthesis and growth. 

First, we observed that *LhGLK1* not only enhanced leaf chlorophyll content but also promoted chloroplast formation in non-green tissues. Interestingly, we did not observe chloroplast in root epidermis cells. Chloroplasts were distributed mainly in the middle of the primary root and petal vascular bundle. This is not a new discovery, as it was previously observed in many similar studies [3]. We assume that other factors prevent chloroplast formation based on cell type and its specific gene expression pattern. In a similar study of overexpressing *AtGLKs* in *Arabidopsis*, chlorophyll autofluorescence was also tested in the root of WT, which had fewer chloroplasts in comparison to transgenic lines [26]. In our study, we did not test any chlorophyll autofluorescence in root cells of WT. The authors of the aforementioned study performed chlorophyll autofluorescence when plants were cultured for 21 days, but in our study, we used 8 days. This situation indicated that the mature root of Arabidopsis has the capacity to synthesize chlorophyll and chloroplasts when cultured in light conditions, but not the seedling root. Overexpressing *LhGLK1* in *Arabidopsis* may bring this event forward.

Next, GLK target genes are mainly associated with the photosystem complex and chlorophyll synthesis. Due to OE lines exhibiting a dark-green phenotype and containing more chlorophyll content, we conducted qRT-PCR to test the transcription levels of GLK target genes in rosette leaves, which were previously reported by ChIP in Arabidopsis [20]. In opposition to what was expected, the transcription levels of most tested genes were not up-regulated in OE lines when compared to the control lines in the leaf. That was consistent with previous studies in Arabidopsis [17,20]. Transcription levels of most AtGLK1 downstream target genes have no remarkable difference in comparison to WT in a microarray analysis of a GLK1-overexpressing line, which is based on a wild-type background. Particularly, the *glk1 glk2* double-mutant background is opposite to the above situation [20]. When *AtGLK1* is overexpressed in the *glk1 glk2* double mutant, nearly all transcription levels of downstream genes were significantly up-regulated. We believe that a complicated regulation pathway in the WT background may alleviate the effect of overexpressing *LhGLK1.* Mark T. Waters et al. provided a detailed explanation [20]. But we observed ectopic chloroplasts biogenesis in root of transgenic lines, while no chloroplast in WT. All things considered, we assumed that constitutive overexpression may have a significant impact on the root. As expected, the expression levels of tested genes were up-regulated in the root. This study is partly consistent with the above study and explanation.

Finally, a previous experiment about overexpressing *AtGLK1* in Arabidopsis showed that the efficiency of photosystem II (ΦPSII) and photochemical quenching (qP) of transgenic lines were decreased in comparison to WT [7]. At the same time, the shoot biomass of *AtGLK* overexpressing lines was also decreased, which is opposite to our study. We presumed that this was the result of heterologous expression or the transcription level of *LhGLK1*. In their study, there was a great discrepancy between WT and OE lines in rosette fresh weight and leaf area. Normal biomass accumulation was dramatically decreased in OE lines, which was consistent with the data of qP and ΦPSII. Conversely, we did not observe a markedly suppressed value in rosette leaf area in overexpressing *LhGLK1* lines at the same time, besides the late-flowering phenotype. Furthermore, we do not suppose that the great discrepancy between these two similar experiments was entirely caused by culture condition. We know that ectopic overexpression of maize *ZmGLKs* enhanced rice’s photosynthetic intensity and grain field in rice. Similar to the above situation, other similar research showed that excessive expression of *ZmGLKs* lines generated fewer seeds weight when compared with WT in rice, in which *ZmGLKs* were driven by a mild promoter [22,24]. Moreover, the culture conditions may have an impact on the effect of overexpressing GLK genes. Differences between overexpressing *ZmGLKs* lines and WT may appear when they are cultured under fluctuating light conditions, even though there is no discrepancy in stable light conditions. Overexpressing *ZmGLKs* may alleviate photoinhibition when plants are cultured in strong light or fluctuating light intensity conditions. According to studies in Arabidopsis, the function of AtGLKs primarily influences the genes related to light harvesting and chlorophyll biosynthesis, instead of photosynthetic genes. Consequently, we assume that the overexpression of *LhGLK1* gene indirectly influences photosynthesis in Arabidopsis.

In conclusion, we found that heterologous expression of *LhGLK1* in Arabidopsis caused chloroplast and chlorophyll biosynthesis in the root and rosette leaf, respectively, which is consistent with the function of GLK. We proved that overexpression of *LhGLK1* in the root drove the expression of genes related to photosystem and chlorophyll biosynthesis. In addition, leaf chlorophyll fluorescence in qP and ΦPSII increased with more biomass accumulation of rosette leaf. We still do not have any detailed explanations about the cause of changes in chlorophyll fluorescence parameters. The application of GLK may enhance timber yield and grain yield in practical production. Changes in leaf color may lead to new varieties of ornamental plants.

## 4. Materials and Methods

### 4.1. Plant Materials and Growth Conditions

All experimental *Arabidopsis thaliana* is Columbia ecotype-0 (Col-0). Wild-type (Col-0) and T3 generation transgenic lines seeds were cultured on 1/2 MS medium without antibiotic selection. After 3 days of vernalization, all seeds were germinated in an illumination incubator for 8 days. Then, all genotype plants were transplanted onto soil. All days mentioned in the article refer to the days cultured on soil after 8 days of germination. Growth conditions are 23 °C with a long day 16-h light/8-h dark cycle and 70% humidity. Light intensity at 6500 lux. 

### 4.2. Generation of Overexpressing LhGLK1 Lines

To generate the *p35S:LhGLK1* vector, total RNA from the leaves of 90-day-old *Liriodendron* hybrid seedling was extracted and used for *LhGLK1* cDNA synthesis. Then, *LhGLK1* cDNA was cloned by Phanta DNA Polymerase (Vazyme, P505-d1/d2/d3, Nanjing, China) using gene-specific primer pairs. *LhGLK1* cDNA was then connected to the pBI121 vector by the method of homologous recombination. The *p35S:LhGLK1* vector was transformed into the *Agrobacterium* strain GV3101 for floral dip transformation based on *Arabidopsis thaliana* Columbia-0 (Col-0). Transgenic plants were screened on 1/2 MS medium with kanamycin (50 mg/L) until generation T3. DNA of all transgenic plants was extracted for PCR to confirm the positive plant using T-DNA and gene-specific primer pairs.

### 4.3. Identification of the LhGLK Genes

To identify the *LhGLK* genes, we downloaded GLK1 and GLK2 protein sequences of *Arabidopsis thaliana*, *Oryza sativa*, *Zea mays*, *Malus domestica* and *Populus tomentosa* from Phytozome v13 (https://phytozome-next.jgi.doe.gov/) (accessed on 6 December 2023). These were used as queries to blast against the protein database of *Liriodendron*. The candidate sequences were then aligned with the mentioned GLK genes by DNAMAN 6.0, followed by phylogenetic analysis with the neighbor-joining (NJ) method (1000 bootstrap replicates).

### 4.4. Transmission Electron Microscopy

Cut the rosette leaves (30 day) into 1 mm wide pieces with a blade, fix them in front of 4% glutaraldehyde (prepared with 0.2 Mol phosphate buffer pH 7.2), and permeate with weak vacuum. After cleaning with phosphate buffer for 3 times (20 min each time), fix with 2% *w*/*v* osmium tetroxide (prepared with 0.2 Mol phosphate buffer pH 7.2) and let these stay overnight. After cleaning 3 times with phosphate buffer (20 min each time), dehydrate with 30%, 50%, 70%, 90% concentration of acetone step by step, dehydrate with 100% acetone twice, for 30 min each time at each stage. The embedding agent is infiltrated step by step, embedded, and polymerized overnight in the temperature range of 37 °C–45 °C–60 °C in the incubator. Us RMC ultra-thin microtome for ultra-thin section, section thickness 50–70 nm. Dye with 0.5% *w*/*v* uranium acetate, 0.2% *w*/*v* lead citrate, rinse with deionized water. Observe and photograph the stained sections with a JEM-1400 transmission electron microscope (JEOL, Tokyo, Japan).

### 4.5. qRT-PCR Analysis

*Liriodendron* hybrid flower tissues derived from an adult tree in Nanjing Forestry University (Nanjing, China) were used. *Arabidopsis* tissues came from the largest rosette leaves of experimental plants. The roots were derived from seedlings cultured on medium for 20 days. All tissues were saved in liquid nitrogen. Then, total RNA was isolated by RNA extraction kit (Promega, LS1040, Shanghai, China). RNA was reversed into cDNA to conduct Quantitative real-time PCR (qRT-PCR) using Vazyme AceQ qPCR SYBR Green Master Mix (without ROX) (Q121-02) on a LightCycler 480 II (Roche, Basel, Switzerland) and specific primer pairs in Table S1. The result was normalized with *Liriodendron* hybrid 18S and *Arabidopsis Actin-2* as a reference, respectively.

### 4.6. Western Blot Analysis

The total protein of leaf (30-day) was extracted as described [37]. Then, 10 µg protein of each line was used for polyacrylamide gel electrophoresis, and electrotransferred onto nitrocellulose membranes. Then, the specific antibodies of Lhca1 and Lhcb3 were used for reacting with a protein band, which was detected by chemiluminescence reagent. ImageJ 1.8.0 was used to calculated gray value of band. Antibodies and relative reagent were applied by ORIZYMES (Shanghai, China).

### 4.7. Chlorophyll Content and Measurement 

The materials came from rosette leaves of all lines when seedlings were cultured on soil for 15 days. Rosette leaves chlorophyll of each genotype was extracted complying with the protocol of the test kit (NJJC, A147-1-1,Nanjing, China). Leaves were ground using quartz sand. Then, chlorophyll was extracted by a mixture of acetone and ethanol (1:1). Chlorophyll content was measured on a microplate reader (Molecular Devices, SpectraMax M3, Sunnyvale, CA, USA) at 645 nm and 663 nm. Chlorophyll a and chlorophyll b were calculated as described [38]. 

### 4.8. Chlorophyll Autofluorescence Analysis 

Chlorophyll autofluorescence between 660 and 700 nm was detected under 488 nm laser excitation and merged with the bright field based on confocal Zeiss LSM 800 (Oberkochen, Germany). Smart setup mode was used to choose laser at 488 nm, pinhole 40 μm, Master Grian at 660 V, Digital Offset at 0, and Digital Gain at 1.0. The image was taken with a 20× objective lens. Tile mode was used for image stitching. All steps comply with a protocol [39]. Root tissues from T3 generation seedlings were cultured on 1/2 MS medium without antibiotic for 8 days under long-day conditions. Primary roots from the shoot about 2 cm were tested. Petals were extracted from flowers at the top region of the stem, which were cultured on soil for 25 days. At least 5 biological replicates (roots or petals) for each genotype were used in the assays. 

### 4.9. Flowering Time and Rosette Leaf Mass Measurements

Flowering time was determined when the first flower was opened or reached to phrase 6.00, according to the method of Douglas C. Boyes [40]. At the same time, the number of rosette leaves was counted. Total rosette fresh mass of each genotype was measured by an analytical balance when experimental plants were cultured on soil for 15 days. Dry weight was measured after drying for 7 days at 70 °C.

### 4.10. Chlorophyll Fluorescence Measurement

The 4th real rosette leaf of each genotype was used to conduct chlorophyll fluorescence measurement by DUAL-PAM/F (Walz, Effeltrich, Germany) when plants were cultured on soil for 15 days. All transgenic plants were placed in a dark environment for 20 min. Dual channel mode Fluo + P700 and SP-analysis was used for measuring. Each pulse was spaced 30 s apart for a total of five minutes. Values were derived from light-induced curves. The results were automatically output by the instrument. The measurement mode complied with the advice of the engineer.

## Data Availability

All data generated or analyzed during this study are available within this article or upon request from the corresponding author.

## Supplementary Materials

The following supporting information can be downloaded at: https://www.mdpi.com/article/10.3390/ijms25136968/s1.
