# Supplementary material for: Overexpression of Liriodendron Hybrid LhGLK1 in Arabidopsis Leads to Excessive Chlorophyll Synthesis and Improved Growth
Source: Int J Mol Sci. 2024 Jun 26;25(13):6968. doi: 10.3390/ijms25136968 (PMC11241243; doi:10.3390/ijms25136968)
Supplement: Supplementary file 1 [file ijms-25-06968-s001.zip › Supplymental Figures.pdf]

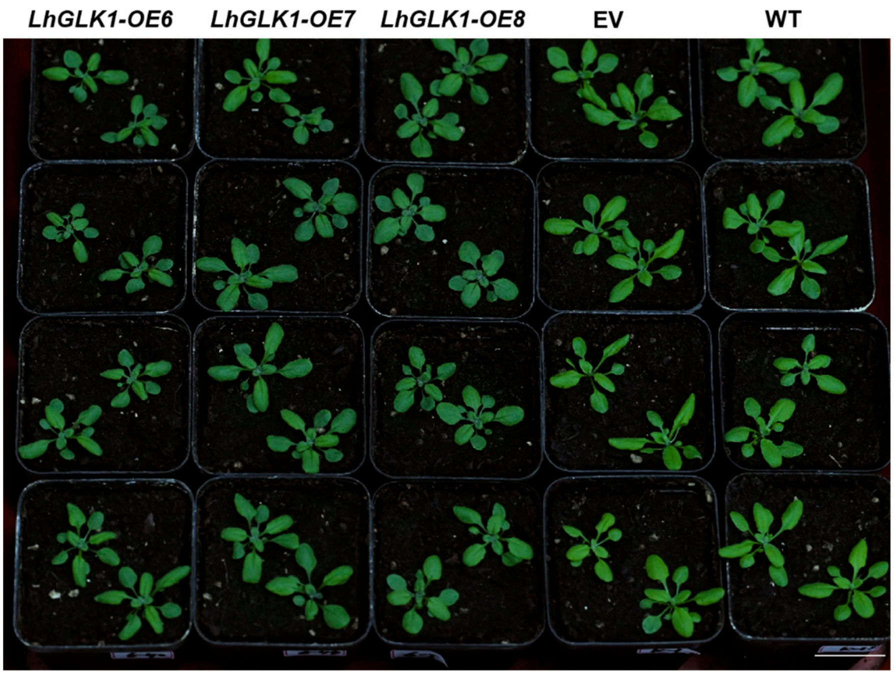

Supplemental Figure S1. The phenotype of dark green leaves in the *LhGLK1* overexpression lines, empty vector (EV) and wildtype (WT). Scale bar = 5 cm.

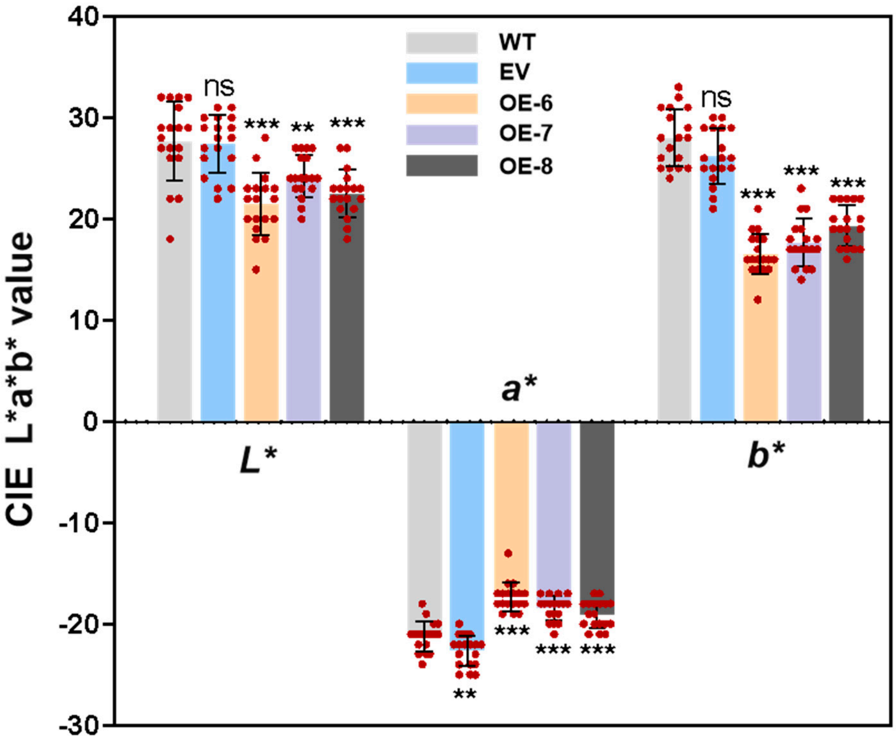

Supplemental Figure S2. Vision disparity measured by CIE into three aspects L, a, b in LhGLK1 overexpression lines, empty vector lines and wildtype Arabidopsis.

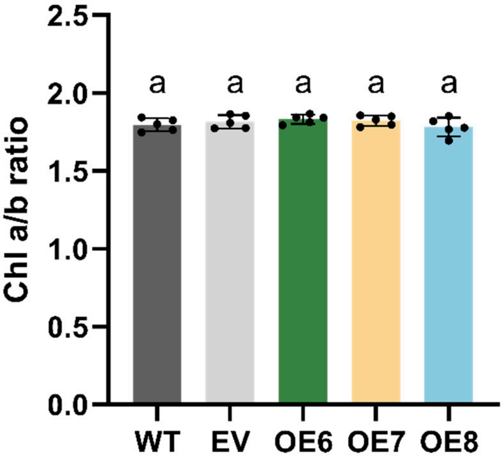

Supplemental Figure S3. Chlorophyll a and chlorophyll b ratio of WT, EV and transgenic lines.
